# Supplementary material for: High-Cholesterol Diet Decreases the Level of Phosphatidylinositol 4,5-Bisphosphate by Enhancing the Expression of Phospholipase C (PLCβ1) in Rat Brain
Source: Int J Mol Sci. 2020 Feb 10;21(3):1161. doi: 10.3390/ijms21031161 (PMC7038105; doi:10.3390/ijms21031161)
Supplement: Supplementary file 1 [file ijms-21-01161-s001.zip › ijms-683810-supplementary.pptx]

## Slide 1
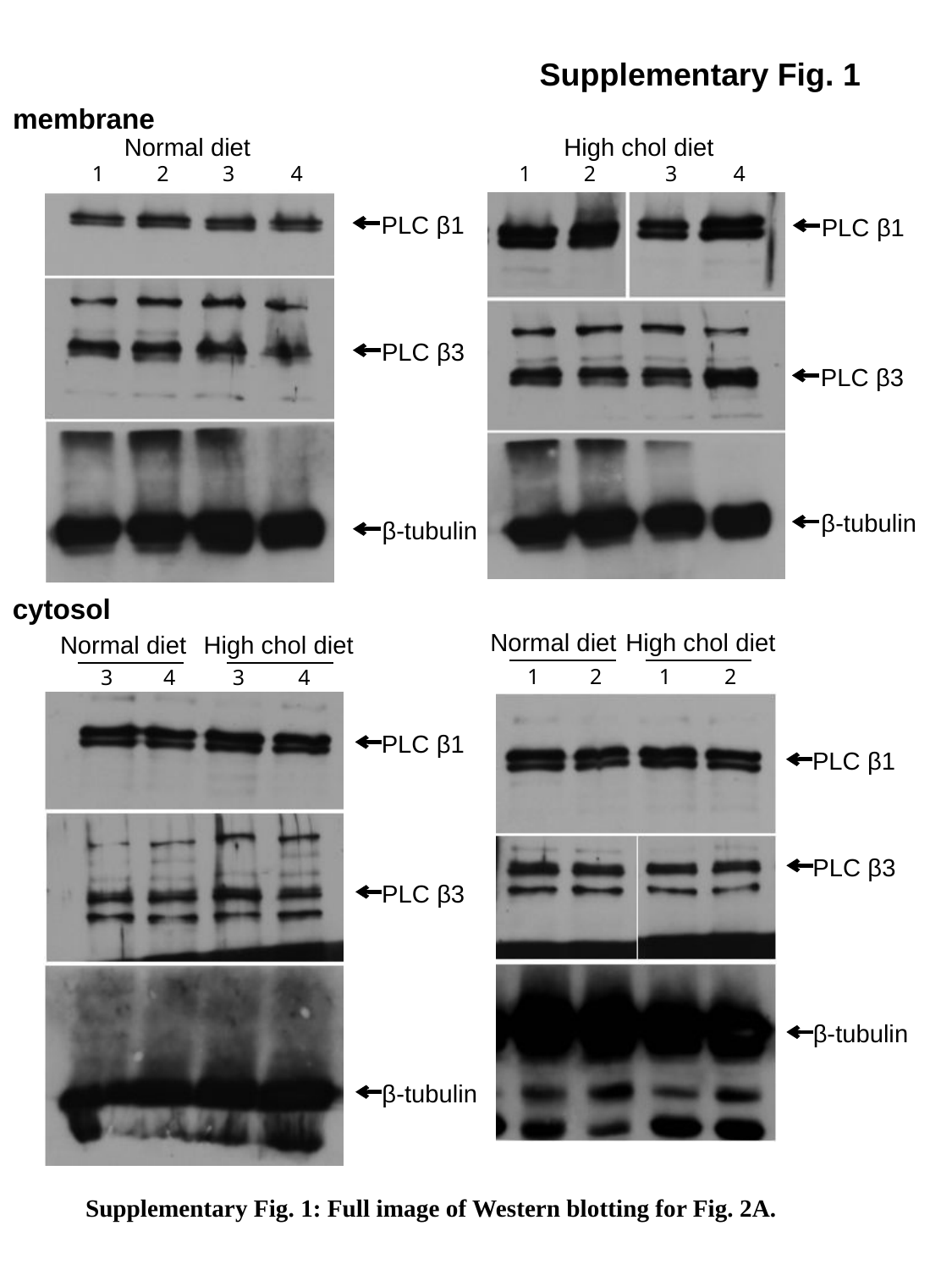

Supplementary Fig. 1
membrane
Normal diet
1
2
3
4
High chol diet
1
2
3
4
PLC β1
PLC β1
PLC β3
PLC β3
β-tubulin
β-tubulin
cytosol
Normal diet
High chol diet
1
2
1
2
Normal diet
High chol diet
3
4
3
4
PLC β1
PLC β3
β-tubulin
PLC β1
PLC β3
β-tubulin
Supplementary Fig. 1: Full image of Western blotting for Fig. 2A.

## Slide 2
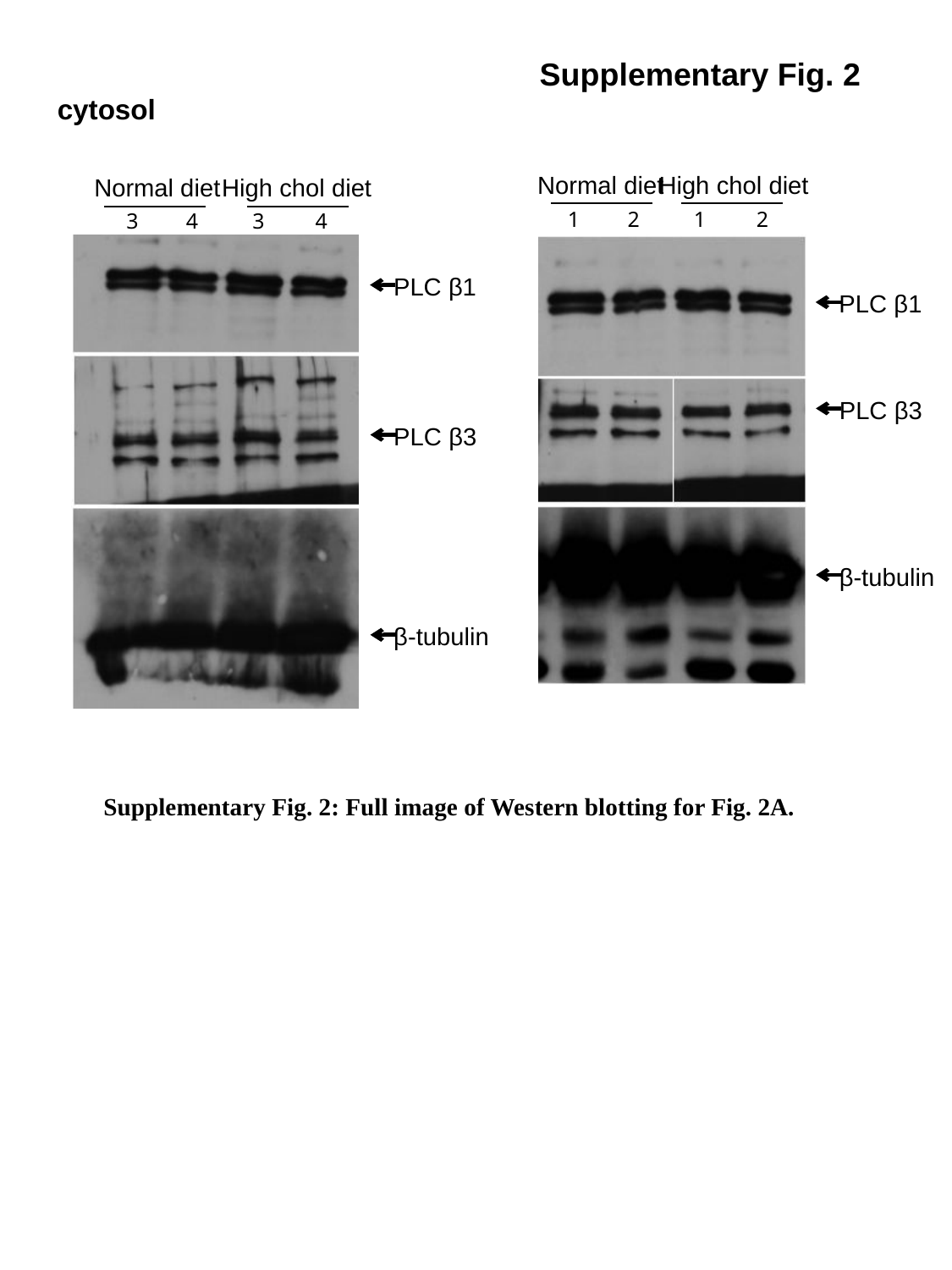

Supplementary Fig. 2
cytosol
Normal diet
High chol diet
1
2
1
2
Normal diet
High chol diet
3
4
3
4
PLC β1
PLC β3
β-tubulin
PLC β1
PLC β3
β-tubulin
Supplementary Fig. 2: Full image of Western blotting for Fig. 2A.

## Slide 3
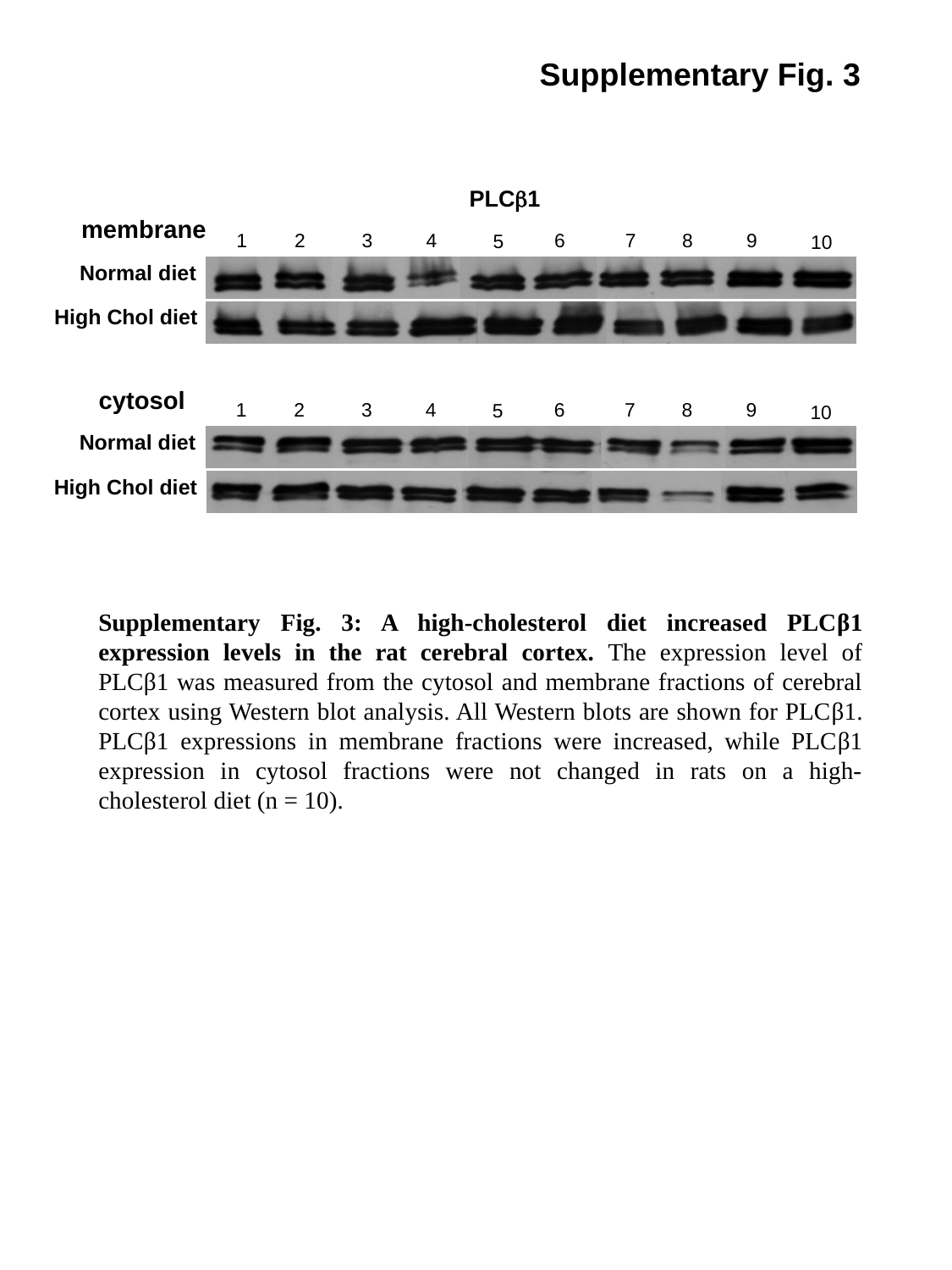

Supplementary Fig. 3
PLCb1
membrane
1
2
3
4
6
7
8
9
5
10
Normal diet
High Chol diet
cytosol
1
2
3
4
6
7
8
9
5
10
Normal diet
High Chol diet
Supplementary Fig. 3: A high-cholesterol diet increased PLCβ1 expression levels in the rat cerebral cortex. The expression level of PLCβ1 was measured from the cytosol and membrane fractions of cerebral cortex using Western blot analysis. All Western blots are shown for PLCβ1. PLCβ1 expressions in membrane fractions were increased, while PLCβ1 expression in cytosol fractions were not changed in rats on a high-cholesterol diet (n = 10).
